# Atlantic Bluefin Tuna: A Novel Multistock Spatial Model for Assessing Population Biomass

**DOI:** 10.1371/journal.pone.0027693

**Published:** 2011-12-09

**Authors:** Nathan G. Taylor, Murdoch K. McAllister, Gareth L. Lawson, Tom Carruthers, Barbara A. Block

**Affiliations:** 1 Fisheries Center, University of British Columbia, Vancouver, British Columbia, Canada; 2 Department of Biology, Woods Hole Oceanographic Institution, Woods Hole, Massachusetts, United States of America; 3 Hopkins Marine Station, Stanford University, Pacific Grove, California, United States of America; University of California San Diego, United States of America

## Abstract

Atlantic bluefin tuna *(Thunnus thynnus)* is considered to be overfished, but the status of its populations has been debated, partly because of uncertainties regarding the effects of mixing on fishing grounds. A better understanding of spatial structure and mixing may help fisheries managers to successfully rebuild populations to sustainable levels while maximizing catches. We formulate a new seasonally and spatially explicit fisheries model that is fitted to conventional and electronic tag data, historic catch-at-age reconstructions, and otolith microchemistry stock-composition data to improve the capacity to assess past, current, and future population sizes of Atlantic bluefin tuna. We apply the model to estimate spatial and temporal mixing of the eastern (Mediterranean) and western (Gulf of Mexico) populations, and to reconstruct abundances from 1950 to 2008. We show that western and eastern populations have been reduced to 17% and 33%, respectively, of 1950 spawning stock biomass levels. Overfishing to below the biomass that produces maximum sustainable yield occurred in the 1960s and the late 1990s for western and eastern populations, respectively. The model predicts that mixing depends on season, ontogeny, and location, and is highest in the western Atlantic. Assuming that future catches are zero, western and eastern populations are predicted to recover to levels at maximum sustainable yield by 2025 and 2015, respectively. However, the western population will not recover with catches of 1750 and 12,900 tonnes (the “rebuilding quotas”) in the western and eastern Atlantic, respectively, with or without closures in the Gulf of Mexico. If future catches are double the rebuilding quotas, then rebuilding of both populations will be compromised. If fishing were to continue in the eastern Atlantic at the unregulated levels of 2007, both stocks would continue to decline. Since populations mix on North Atlantic foraging grounds, successful rebuilding policies will benefit from trans-Atlantic cooperation.

## Introduction

Atlantic bluefin tuna *(Thunnus thynnus)* is a large, endothermic, and highly migratory member of the tuna family, Scombridae. They can reach a mass of 650 kg and live to be over 32 years old [1]. Historically, its range has encompassed much of the North Atlantic, from the waters off Norway and the Faroe Islands to the South Atlantic and the west coast of Africa. Atlantic bluefin occurrences have been reported from Mauritania [2] and off South Africa [3]. In the western Atlantic Ocean, the species' historic range extended from Canada to Brazil, including the Gulf of Mexico and Caribbean Sea. In the twentieth century, the population appears to have disappeared from the southern part of its range and the North Sea [4].

Recent studies have shown that spatial population structure and movements are more complicated than previously thought. Conventional [5] and electronic tagging [6], [7], [8] studies, as well as genetic [9], organochlorine tracer [10], and otolith microchemistry [11] studies, indicate that three or more populations of Atlantic bluefin tuna exist [12]. Genetic studies indicate that at least two populations spawn in the Mediterranean Sea in summer months [12]. In the Gulf of Mexico, a smaller population spawns in the spring months (April–June). Histological sampling of fisheries catches indicates half of the fish spawned in the Gulf of Mexico are sexually mature at age 12 [13]. This has been corroborated by electronic tagging data for which the mean age of individuals returning to the Gulf to spawn was 11.8 years [7]. In comparison, the age at maturity in the eastern Mediterranean population is considered to be 4 years [14]. However, fish tagged in the western Atlantic that return to breed in the western Mediterranean spawning areas enter at Gibraltar on average at ages 7–9 [7]. Site-directed fidelity has been observed [7], [15] and is hypothesized to maintain genetic structuring [12].

Atlantic bluefin tuna populations are managed by the International Commission for the Conservation of Atlantic Tunas (ICCAT) as western and eastern populations, or stocks, separated by the 45^o^ meridian. Both populations are considered to be overfished [13], [15], [16], and rebuilding policies in the western Atlantic do not appear to have been successful to date. Bycatch of bluefin tuna in areas closed to directed bluefin fishing, such as the Gulf of Mexico, remains problematic [17]. Illegal and underreported catches, due in part to widespread tuna ranching, have been a severe problem in the Mediterranean Sea, and scientists have had to adjust reported catches using Japanese import records for assessments [13]. For example, in 2006, the reported eastern Atlantic and Mediterranean catches were 31 kilotonnes (kt), but import records suggested that as much as 54 kt were caught [13].

The determination of whether a stock is overexploited requires the prediction of historical spawning stock biomass (SSB), which has proven difficult to determine for Atlantic bluefin tuna. A central reason is that the multiple Atlantic stocks are mixed on fishing grounds and demonstrate stock-specific movements. In much of the western Atlantic Ocean, biological markers [9]–[11] show that eastern and western Atlantic bluefin stocks co-occur. Tagging studies indicate that large-scale migrations of 7400 km or more routinely take place across the ICCAT stock boundary and between the western and eastern Atlantic and the Mediterranean Sea by bluefin tuna of all ages [6], [7], [18]. In addition, recent results from tagging, genetics, and microchemistry markers demonstrate stock-specific seasonal and/or ontogenetic movements [6], [7], [18], [19]. Ontogeny and population origin influence which areas a bluefin tuna utilizes in the North Atlantic, so that in any given area, age or season, there can be different proportions of each population. Mixing of populations compromises the accuracy of the single-stock models that are currently used to determine SSB declines because some catches have been attributed to the incorrect stock of origin.

Because ICCAT does not routinely consider population mixing in assessments, it may not effectively understand or control fishing mortality on individual Atlantic bluefin tuna populations. Even though a mixed-stock assessment model exists [20], [21], current ICCAT bluefin tuna assessments primarily use single-stock virtual population analysis (VPA) [22]. The single-stock VPA assumes that all bluefin tuna catches west of the 45^o^ meridian are from the western spawning population, and that all fish to the east of this longitude are from the eastern population. Failure to accurately account for seasonal movements and ontogenetic distinctions, as well as western and eastern stock mixing, can therefore compromise the reliability of current and future population size estimates. In turn, projections of the effects of various policy actions are likely to be unreliable.

In this paper, we provide a new seasonally and spatially explicit fisheries model that incorporates population mixing in an effort to improve our capacity to assess Atlantic bluefin tuna population sizes. This is a multi-stock age structured tag integrated assessment model that we refer to as MAST. This population dynamics model runs on quarterly intervals, incorporates catch data from 1950 to 2008, and is fitted to (1) age-composition landings data from 1960 to 2008; (2) 29 stock-trend time-series derived from commercial and research catch-per-unit-effort (CPUE) series [13]; (3) ICCAT conventional (“spaghetti”) tagging data [5]; (4) archival and pop-up satellite archival tag data [7]; and (5) published otolith microchemistry data [11]. The model assumes time-invariant gear selectivity and the reporting rates for conventional tags documented by Kurota *et al*. [23]. Using this model, we applied Bayesian integration using Markov Chain Monte Carlo Simulation (MCMC) to account for uncertainties in model parameters and predictions of spawning stock biomass and fishing mortality rates from 1950 to the present.

We evaluated the rebuilding efficacy of management scenarios that capture a range of alternatives previously considered for Atlantic bluefin tuna management. We examined five cases: near-complete fisheries closures that could have occurred under a Convention for the International Trade in Endangered Species (CITES) listing [15]; 2010 ICCAT quotas, with and without a Gulf of Mexico spawning area closure with catch redistributed; a scenario that assumed that actual eastern catches were double the 2010 ICCAT quotas; and, finally, a scenario of very high eastern Atlantic and Mediterranean catch levels that occurred from the late 1990s to 2007.

## Methods

### Modeling Approach

The Multistock Age-Structured Tag-integrated assessment model (MAST) is a mixed-stock, seasonal, and spatially explicit statistical catch-at-age model that can be fitted to relative abundance indices, age proportions, and otolith microchemistry, as well as to conventional and electronic mark-recapture data. The model was written and fitted to data using the software AD Model Builder, which is freely available from www.admb-project.org (ADMB Project 2009). The model and statistical fitting procedure are described in detail in the online Text S1. We characterized parameter uncertainty using Markov Chain Monte Carlo simulation.

The MAST model consists of four major components:

1. Initialization of the model based on steady-state conditions (unfished numbers and biomass) given the model's parameters;
2. Updating the state variables (numbers and biomass at age in each area);
3. Relating the state variables to observations on relative abundance, age-composition information, and mark-recapture observations; and
4. Evaluating the probability of model parameters given the data.

We provide a description of each model component in the main text, and refer readers to the Model Description in the online Text S1 for further detail.

We defined five geographic areas for quantifying movement dynamics: the Gulf of Mexico, which we assume is the western-stock spawning area; the Gulf of St. Lawrence, which we assume contains primarily western-stock fish [11]; the western and eastern Atlantic Ocean, which we assume to be mixed-stock areas; and the Mediterranean Sea, which we assume is the eastern-stock spawning area. We used these areas because they are either mixed-stock areas in the eastern and western Atlantic Ocean basins that have historical importance at ICCAT, or because they appear to be nearly exclusively western-stock (the Gulf of Mexico and Gulf of St. Lawrence) or eastern-stock (Mediterranean Sea) areas. Figure S1 shows the model areas and the electronic tag geoposition data. By including the Gulf of St. Lawrence as a distinct area, additional tagging data and an additional CPUE abundance index can be used in modeling the population dynamics of the western stock [11]. MAST models western-stock fish as moving between the Gulf of Mexico, the Gulf of St. Lawrence, and the western and eastern Atlantic (corresponding to area indices 1–4). MAST models eastern-stock fish as moving between the Mediterranean Sea and the eastern and western Atlantic (area indices 3–5).

We parameterized movement matrices using gravity models for the model-fitting base-case. The probability of fish moving from one area to another is defined in terms of a movement matrix *µ*. Each movement matrix consists of rows representing area of origin and columns representing the destination area. Each row element of *µ* therefore represents the probability of fish moving from area *j* (rows) to area *j*' (columns); each row represents a probability vector *υ,* where *υ* = (*υ*_1_, *υ*_2_, …, *υ*_n_) and *Συ* = 1. Here we estimated a single propensity of fish to stay in a given area—that is, “gravity” (the diagonal elements of *µ*)—which is assumed to capture the attractiveness of that area relative to the areas associated with the off-diagonal elements. These latter elements are given as (1-*µ_j,j_*)/(*n*–1), where *n* is the number of stock areas for stock *i*. An alternative to the gravity model is the bulk-transfer model, in which the full matrix of movement probabilities is estimated. Some biological detail is lost in using this gravity parameterization; the main advantage is that it substantially reduces the number of estimated parameters compared with the bulk-transfer case. We discuss the bulk-transfer parameterization and the sensitivity of the model in the online Text S1.

For Atlantic bluefin tuna, there is strong seasonal and ontogenetic dependence of movement rates, where fish of different ages use different habitats during the year for foraging or spawning [6], [7], [18]. To account for these phenomena, we modeled quarterly time-steps and two age-groups: 0–7 and 8+ We assumed that movement transitions to spawning areas (the Gulf of Mexico for the western stock, and the Mediterranean Sea for the eastern stock) during the spawning quarter were given by the maturity-at-age schedule.

The MAST model uses the management-oriented approach [28], meaning that the model is initialized using maximum sustainable yield *(MSY)* and the fishing rate that produces maximum sustainable yield *(F_msy_)*. Under this formulation, *MSY* and *F_msy_* are the leading estimated parameters. Then, using estimated gear selectivity as well as input growth [1], mortality, and maturity parameters, we derived the recruitment compensation parameter κ [29], initial numbers, and initial biomass *B_0_*. Maturity-at-age schedules were based on [30] for the western stock and [31] for the eastern stock. The model was parameterized with initial numbers-at-age in the spawning area and then run for 25 years to allow the model to equilibrate between areas (see Tables S1–S7).

Initial numbers-at-age for each stock (see Tables S1–S7) were updated in each time-step (i.e., the next quarter) according to natural and fishing mortality, as well as migration parameters. Age-zero recruits were predicted by a Beverton-Holt stock recruit function (Eq. 25, Table S5). Details of the state dynamics are described in the online Text S1.

### Parameter Estimation

We estimated the parameters that define the model by fitting predicted observations (component 3) to observed data. The modeling procedure starts with initial parameter values (see Tables S1–S5) that define the state variables, then proceeds through the state dynamics (Tables S4 and S5), where state variables such as numbers-at-age for each stock are updated at each time-step. Ultimately, the model calculates the statistical objective function value, which represents the probability of the model given the data (Table S6). Parameters are estimated using a conventional nonlinear optimization procedure. AD Model Builder was used to implement the model.

For all data types except electronic tag data, we used conventional statistical likelihoods. We fitted relative abundance indices using Walters and Ludwig's [32] formulation (see Tables S5 and S6). We fitted otolith microchemistry using binomial likelihoods, and conventional mark-recapture data using negative binomial likelihoods (Tables S4 and S5). If the stock-of-origin for a given cohort was known through a cohort's area of visitation or marking, then that cohort's survival and movement dynamics were modeled according to the movement probability matrix for that stock. However, for 85% of cohorts (Table S10), stock of origin was unknown. In these cases, the likelihood was computed twice; that is, using movement probability matrices from western and eastern stocks (Eq. 1.38, Table S5), with likelihood weights given by the ratio of vulnerable numbers of stock *i* to total vulnerable numbers in that area at that time.

For electronic tag data, we used discrete, state-space likelihoods. We modeled the state of tags through discrete states at each model time-step [33] (see Eqs. 1 and 2 in Table S2). We assumed that the tag was attached to a live fish in area *j*; captured on a fishing vessel; attached to a fish that died of natural causes; or shed from the fish (Table S8). For electronic tags, equations describing state transitions are listed in Table S8, and parameters for electronic tag observation probabilities *p(y_t_|s_t_)* are given in Table S9. When modeling tag tracks, capture probabilities represent the probability of obtaining a geoposition for a particular tag type. In the case of pop-up satellite archival tags, there are complete tag tracks (i.e., spatial positions at each quarter), so these observation probabilities are 1. For archival tags, however, not all tag tracks are complete; there can be missing geolocations at times between the last geolocation recorded by the tag and the location given by the vessel position at time of recapture. In these cases, we estimated a single observation probability parameter for archival tags (Table S9) that represents the proportion of time between the release of the tag and its recovery in which it was possible to determine the geoposition of the tag.

### Data

We used catch data from the 2010 ICCAT CATDIS database (www.iccat.int). CATDIS is the official database that contains catches in 5×5 degree grid squares, by quarter and gear. We separated catches into four gear categories: longline, purse seine, bait boat, and other. For each catch record, an area was assigned according to Fig. S1. The input data for MAST consisted of total catches by fleet, area, and quarter from 1950 to 2008 (Fig. S2). To account for large catch underreporting from 1998 to 2007 in the Mediterranean Sea, we inflated catches reported in the Mediterranean. We used the same procedure as RUN 14 of the 2008 ICCAT stock assessment, where total eastern catches were assumed to be 50,000 metric tonnes (mt) from 1998 to 2006 and 60,000 mt in 2007 [13]. At the time of writing, catch data for 2008 and 2009 were not yet available, so we assumed that the total eastern and western catches in these years were the recommended quotas. This may be a reasonable assumption, since there is evidence that compliance has improved considerably with ICCAT's introduction of a vessel monitoring system in 2008 [13].

We aggregated conventional tag data into cohorts *(h)* for fish that had the same assigned age and were captured in the same quarter; these data are available in ICCAT's conventional tag database (www.iccat.int) and are summarized in [5]. (We used the version of the database updated in September 2009.) The data were filtered to remove incomplete records that were missing size or location data at either release or recapture. The filtered conventional tag data set consisted of 47,439 releases that were distilled into 1732 cohorts. Of these, 125 tag cohorts were assigned to the western stock and 142 to the eastern stock, and 1465 were unknown (Table S10). Details of how tagged fish (both electronic and conventional) were assigned to stocks and age-groups can be found in the online Text S1.

Between 1996 and 2008, a total of 968 bluefin tuna were electronically tagged with internally implanted archival tags and/or externally attached pop-up satellite archival tags at tagging locations along the U.S. East Coast, in the Gulf of Mexico, in the Gulf of St. Lawrence, off Ireland, and in the Mediterranean Sea [6], [7], [18]. The daily geopositions of electronic tags were aggregated to quarterly area assignments. If a tag was reported being in more than one discrete stock area, it was assigned to the area where it spent the greatest proportion of time. Additional details of how satellite geopositions were determined are given in the online Text S1.

We used the commercial CPUE time-series and catch-at-age proportions from the ICCAT assessment document [13]. We used the catch-at-age data from the assessment to define catch-at-age proportions [13] from age 1 to 10+in the western Atlantic (areas 1–3) and eastern Atlantic (areas 4–5), which were based on western and eastern catch-at-age data from 1960–2007 and 1970–2007, respectively. Table S11 is a summary of which CPUE series we used, as well as the corresponding quarters and area for each.

We extracted otolith microchemistry stock-composition data from Rooker *et al.* [11], who divided their data into three age-groups: giant (age 10+), medium (age 5–9), and school (age 4 or younger). They had stock-composition samples for the Mediterranean, Gulf of Mexico, Gulf of St. Lawrence, Gulf of Maine, and the Mid-Atlantic Bight. We fitted the model (see below) to stock-composition ratios from the Gulf of Maine and the Mid-Atlantic Bight only because it was assumed that the Gulf of Mexico and Gulf of St. Lawrence areas were 100% western stock and the Mediterranean was 100% eastern stock. The stock-composition data used in the model are summarized in Table S12.

### Uncertainty and Projections

We computed marginal posterior probability distributions for all estimated parameters using MCMC simulation with six chains. One value was sampled for every ten iterations, and we ran the MCMC until the multivariate posterior scale reduction factor [34] was below 1.05. We present fishing mortality rate reconstructions by area and gear type at the posterior mode; posterior samples of western and eastern bluefin tuna spawning stock biomasses; stock status relative to maximum sustainable yield; and stock composition in mixed-stock areas.

We ran the base-case model with a series of management options, including complete fisheries closures, spatial closures, and other quota options. We chose scenarios to reflect a broad range of possibilities in Atlantic bluefin tuna management. The first scenario (i) represents total closures, which might have occurred with listing under CITES. For the quota scenarios, we assumed future bluefin tuna catches west (W) and east (E) of the 45° meridian from 2010 to 2025 to be: (ii) 1750 mt W/12,900 mt E, with no Gulf of Mexico closure; (iii) 1750 mt W/12,900 mt E, assuming a Gulf of Mexico closure with catches redistributed to the western Atlantic; (iv) if eastern catches continued to be double the current quotas, that is, 1750 mt W/25,800 mt E; and (v) an eastern overfishing case of 1750 mt W/60,000 mt E. This final scenario was intended to capture what might have occurred if Atlantic bluefin tuna catches continued at 2007 levels.

In addition to parameter uncertainty, we examined the sensitivity of the base-case results to a suite of alternative model parameterizations and reporting-rate-prior distributions. The details of each sensitivity case and the corresponding effect of each to key stock status metrics are listed in Table S13, and the effect on conventional tag reporting rates is given in Table S14.

## Results

### Historical Abundance and Exploitation of Atlantic Bluefin Tuna

**Figure 1:**
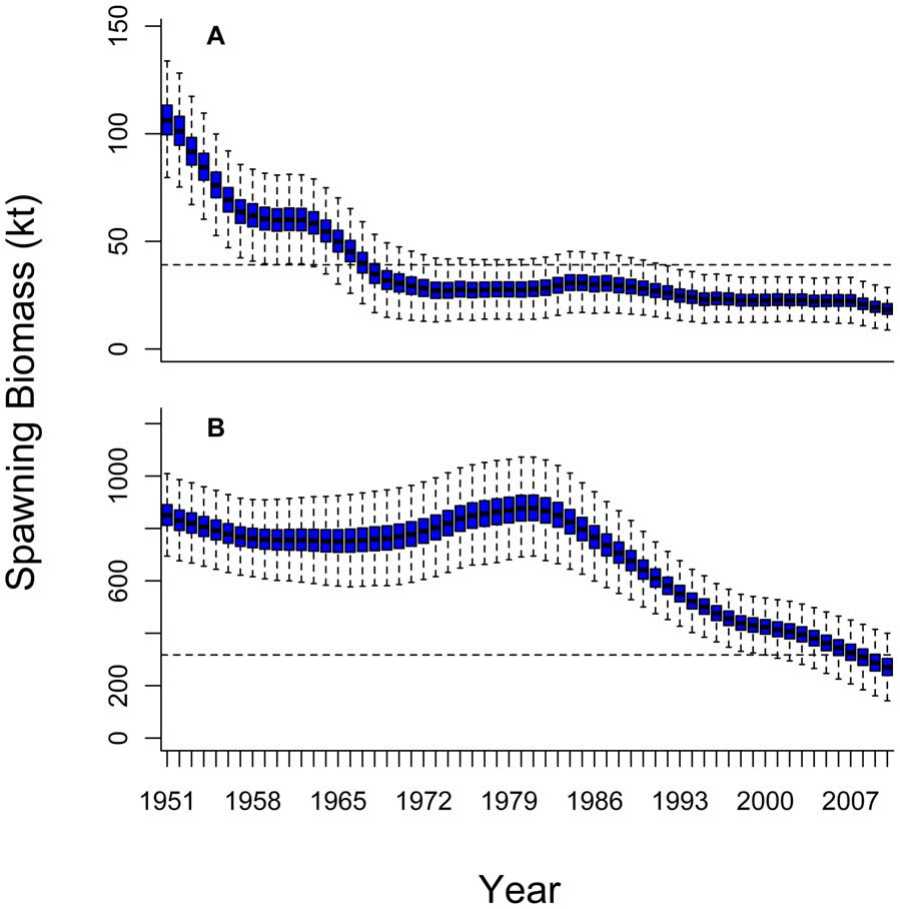
Box plots of posterior samples of the spawning stock biomass (kt) of (A) western and (B) eastern Atlantic bluefin tuna. The horizontal lines within the blue bars represent the posterior median values, the blue bars represent the interquartile values, and whiskers are 1.5 times the interquartile range. The dashed horizontal lines represent the spawning stock biomass that would produce maximum sustainable yield.

MAST estimates the initial stock size *(B_0_)* of the western population to be 100–120 kt, and that of the eastern stock to be 800–900 kt (Figs. 1A and B). The ranges reflect the most credible interquartile ranges. The estimates of the maximum sustainable yield (*MSY*) from which these initial biomasses were calculated are 3.9 and 25 kt for the western and eastern stocks, respectively. Predictions of stock depletion rates relative to 1950 are 17% for the western stock and 33% for the eastern stock.

**Figure 2:**
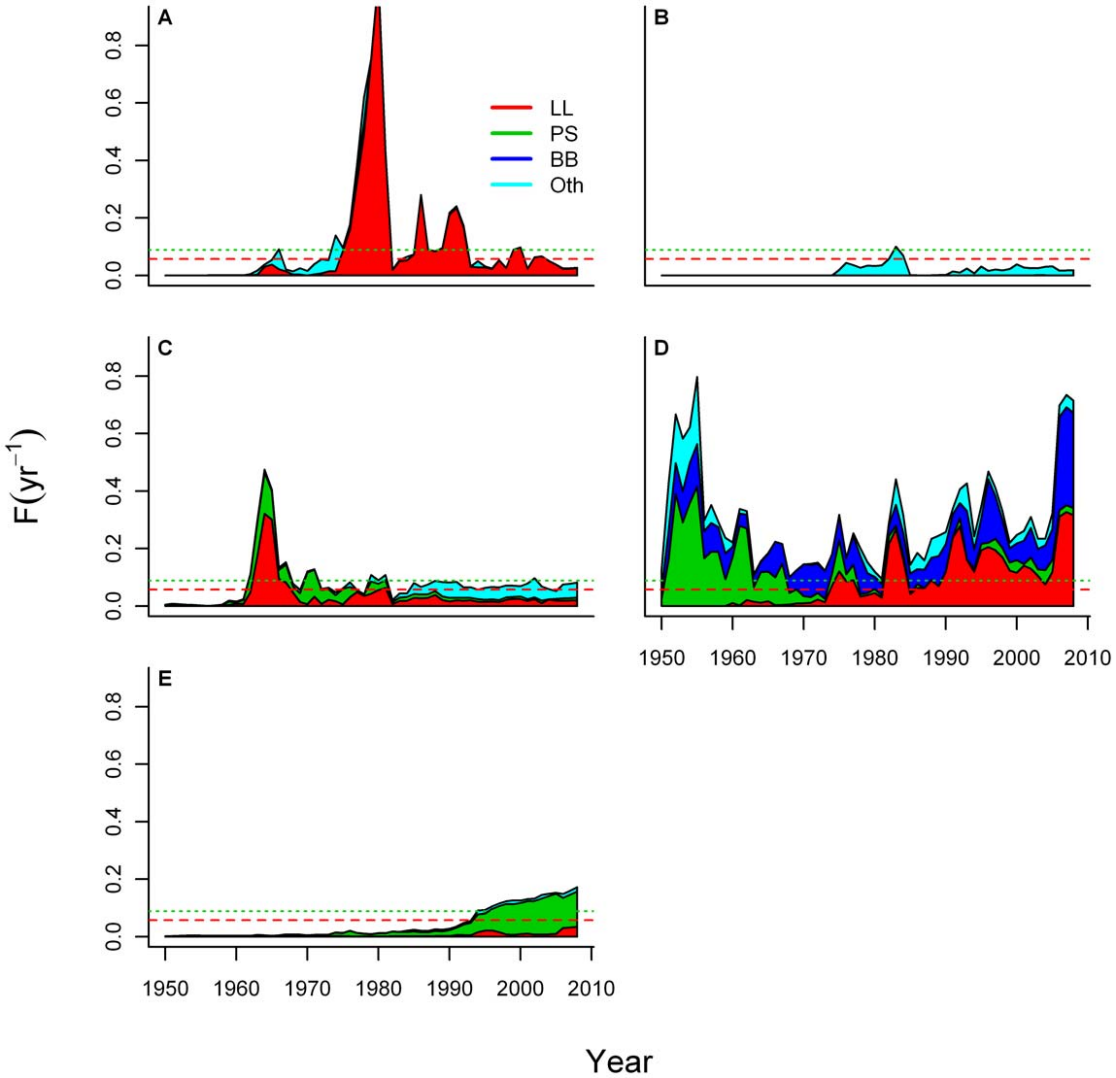
Mean annual fishing mortality rates (yr^-1^) for Atlantic bluefin tuna by longline (LL), purse-seine (PS), bait boat (BB), and other (Oth) gear types in (A) the Gulf of Mexico, (B) the Gulf of St. Lawrence, (C) the western Atlantic Ocean, (D) the eastern Atlantic Ocean, and (E) the Mediterranean Sea. Red and green dotted lines represent *F_msy_* for western and eastern stocks, respectively.

Furthermore, MAST indicates that the western bluefin tuna stock was subject to overfishing and was depleted to below the MSY stock biomass level (*B_msy_*) relatively early in the fishery. Longline and purse-seine fishing in the Northwest Atlantic in the 1960s depleted the stock to levels below *MSY* before 1970 (Fig. 1A). The large annual Gulf of Mexico longline catches (approximately 3–4 kt) that occurred in the 1970s corresponded with high fishing mortality rates (Fig. 2B) on western-stock spawners, which further depleted the stock.

**Figure 3:**
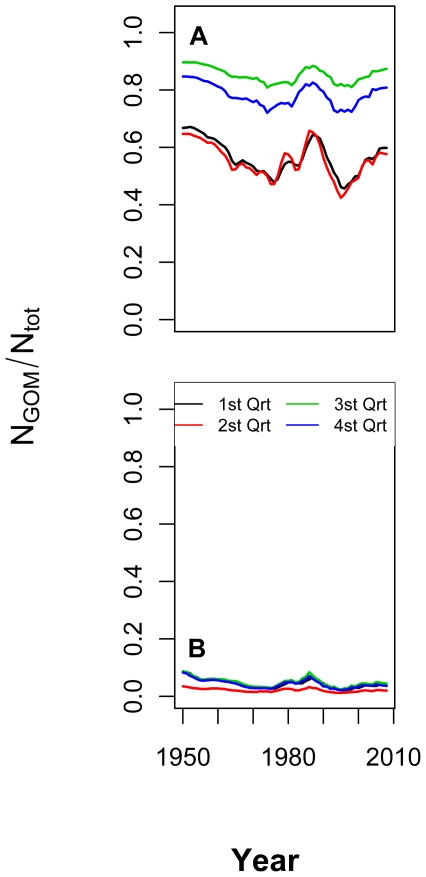
Predicted ratio of the numbers of western Atlantic bluefin tuna to total numbers of tuna from 1950 to 2008 in the (A) western Atlantic Ocean and (B) eastern Atlantic Ocean during the first quarter (black), second quarter (red), third quarter (green), and fourth quarter (blue).

Observed declines in western Atlantic biomass have also been the result of a declining eastern population. The model predicts that the decline of the eastern stock to below *B_msy_* has occurred as recently as the last 10 years (Fig. 1B), owing largely to substantial illegal and unreported catches in the east [13]. Concurrent with the depletion of eastern populations over the last 15 years, the model predicts a steady increase in the ratio of western to eastern fish in the western Atlantic Ocean (Fig. 3A).

The model points to serial, regional depletions as fishing effort has shifted spatially over time. Japanese longlining catches in the Gulf of Mexico in the 1970s were relatively small, but concentrated on a smaller number of western-stock spawners (Fig. 2A). In the western Atlantic, high fishing mortality rates occurred initially from longlining and purse seining, which removed as much as 20,000 mt annually in the 1960s off the coastal United States (Fig. 2A). The Norwegian purse-seine fisheries caught approximately 20,000 mt annually in the early 1960s, exerting mean fishing mortalities of up to 0.8 yr^−1^ until this fishery rapidly collapsed in 1963 [24]. These early fisheries occurred in mixed-stock areas of the western and eastern Atlantic, and the model predicts that a small proportion of Nordic purse-seine catch (Fig. 2D) could have consisted of up to 10% western stock (Fig. 3B). However, the eastern-stock subsidy of western Atlantic bluefin tuna fisheries was substantial, with the western-stock ratio ranging between 50% and 90% western and 10–50% eastern during that period (Fig. 3A). Fishing mortality rates were well above the MSY rate (*F_msy_*) for both stocks in all mixed-stock areas (Figs. 2C and D).

Relative to 1950–1970, eastern Atlantic bluefin tuna catches fell in the 1980s, but then increased again in the 1990s. Since 1990, the bulk of the tuna fishing effort has moved into the Mediterranean Sea in association with purse seining to populate tuna ranches. During this period, the largest Atlantic bluefin tuna catches in history occurred between 1998 and 2007, with maximum catches of approximately 60 kt occurring in 2007 in the eastern Atlantic and Mediterranean Sea [13]. With catches of this magnitude, the model suggests that a very large number of eastern-stock fish, up to 800–900 kt, must have existed in the Mediterranean Sea to have supported such removals. Fishing mortality rates were approximately double *F_msy_* between 1995 and 2008 (Fig. 2E).

### Future Projections of the Atlantic Bluefin Tuna Fishery

**Figure 4:**
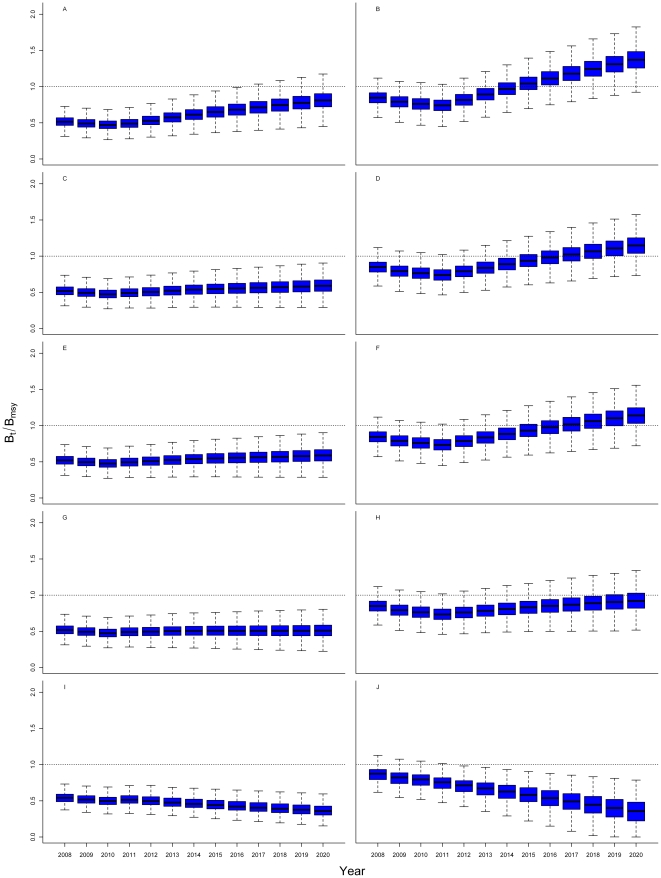
Box plots of the predicted ratio of biomass to the biomass that would produce maximum sustainable yield (B_t_/B_MSY_) under alternative management quotas for western (left column) and eastern (right column) Atlantic bluefin tuna stocks under various scenarios: total fisheries closures (A and B); catches at 1750 mt West and 12,900 mt East, with a Gulf of Mexico closure (C and D); catches at 1750 mt West and 12,900 mt East, with no Gulf of Mexico closure (E and F); catches at 1750 mt West and 25,800 mt East (G and H); and catches at 1750 mt West and 60,000 mt East (I and J). The horizontal lines within the blue bars represent the posterior median values, the blue bars represent the interquartile values, and whiskers are 1.5 times the interquartile range.

MAST predicts that western Atlantic bluefin tuna stock rebuilding depends on eastern Atlantic and Mediterranean catches. If oceanwide catches are zero (scenario i), the model predicts that the western stock has a low probability of being at levels that produce maximum sustainable yield (median *B_2020_/B_msy_* = 0.81, Fig. 4A), but that eastern-stock rebuilding will be much faster (median *B_2020_/B_msy_* = 1.4, Fig. 4B). MAST also predicts that relative to no closure (scenario ii, Figs. 4C and D), western-stock rebuilding will not be faster with a Gulf of Mexico closure (scenario iii, Figs. 4E and F), and eastern-stock rebuilding will be unaffected. However, if eastern-stock rebuilding is slow, or if the stock declines, then western-stock growth must also be slow, or decline, without western quota adjustments to compensate for the loss in the eastern subsidy. In addition, MAST predicts western- and eastern-stock median *B_2020_*/*B_msy_* ratios to be 0.51 and 0.92, respectively, for the double eastern quota (scenario iv, Fig. 4G and H), and 0.36 and 0.35, respectively, for the historical overfishing case (scenario v, Fig. 4I and J). In all cases, the high variability of predicted spawning stock biomasses during the recovery may prevent the benefits of reduced fishing quotas from being statistically detectable for many years.

The redistribution of quota is likely to limit the effectiveness of large-scale closures. Under the Gulf of Mexico closure scenario (iii), the quota tonnage associated with the bycatch and dead or discarded bluefin in the Gulf is redistributed to the western Atlantic. It follows that the predicted landings there would include larger numbers of immature fish of both western and eastern stocks to compensate for their smaller size. The redistribution of quota would not require the Gulf of Mexico fleet to move to western Atlantic fishing areas, because unused quotas could simply be reallocated to other sectors (such as rod and reel) or even other countries through reallocations at ICCAT. There is additional uncertainty over the effects of a Gulf of Mexico closure, because bycatch and dead discard estimates for both inside and outside the Gulf of Mexico are unknown.

## Discussion

We present a fisheries population assessment model that incorporates novel datasets on the spatial and seasonal dynamics of Atlantic bluefin tuna. This is the first assessment to incorporate fine-scale electronic tagging data for this species. Electronic tagging data can provide more precise and reliable seasonal movement and fishing mortality rate estimates than can be obtained from traditional mark-recapture data [23]. Furthermore, satellite tags reveal where tunas go independent of fisheries. By incorporating data that reveal how distinct stocks mix on foraging grounds and separate to breeding grounds in the eastern and western Atlantic, we improve our capacity to capture movement information in the population assessments and understand how movement and mixing may affect management decisions.

The MAST model may be used to conduct fisheries stock assessment and evaluate future management policies. For example, the results of our analysis indicate that eastern and western tuna stocks have experienced systematic declines in the twentieth and twenty-first centuries, with estimated spawning stock biomass depletions of 83% in the west and 67% in the east. The western stock has been severely depleted since the early 1970s, and in the past decade the eastern stock has been subjected to the largest Atlantic bluefin catches since the fishery began. However, rebuilding of the eastern stock is possible in the near future under certain quota scenarios, whereas western-stock rebuilding is predicted to take more than 15 years. MAST results indicate that the incorporation of mixing is critical for understanding historical breeding populations and the efficacy of future quota policies as applied to mixed-stock areas.

These results extend bluefin tuna stock assessments further into the past (i.e., to 1950) than recent ICCAT analyses (i.e., to 1970). Ignoring fishing that occurred before 1950 may, however, bias estimates of depletion levels and biomass reference points. While western Atlantic tuna fisheries began in the middle of the twentieth century, fishing had occurred for several hundred years in the Mediterranean Sea before official catches were recorded beginning in 1950 [24]. This suggests that eastern-stock depletion levels from the unfished state are underestimated. Assuming that the stock was already exploited by 1950 (i.e., not at *B_0_*), unfished biomass, target reference points such as *B_msy_*, and depletion levels relative to *B_0_* could be higher than those estimated by this study, which assumed that the stock was at *B_0_* in 1950. For example, predicted initial stock sizes calculated using deterministic estimates [15] of initial biomass, based on a range of assumptions about recruitment steepness, ranged between 1 and 11.7 million mt. A potential way of accounting for fisheries known to have occurred before reliable catch data were collected would be to consider alternative hypotheses about the initial fishing mortality rate experienced by the population.

The explicit consideration of mixing is likely to improve our understanding of how future Atlantic bluefin tuna populations will respond to alternative management scenarios. Because of the mixed-stock composition of western Atlantic fisheries, the successful rebuilding of the western population is tied to controlling the much larger fishing mortality rates that occur on the eastern stock. For example, continued high fishing mortality rates in the Mediterranean Sea and eastern Atlantic may compromise rebuilding efforts for the western Atlantic population. The converse, however, is not true. The eastern stock is both much larger and much more concentrated in the Mediterranean Sea. ICCAT could potentially increase the chances of successful western-stock rebuilding if it began to model and consider recovery plans [25] for eastern and western populations jointly rather than independently.

Modeling mixed-stock fisheries with complex population dynamics is challenging, and MAST exposes several key sensitivities of the results to model form and parameter inputs. In practice, movements are influenced by ontogeny, stock of origin, and environment, and may have interannual variability [18] or be dependent on oceanographic conditions [26]. In addition, there are alternative forms and time scales of modeling movement dynamics. For example, it is not known how the configuration of model areas and time-steps affects model results. The reliability of both growth and maturity parameters for bluefin are questionable, because samples have come from mixed-stock areas in either the Mediterranean [12] or the western Atlantic [11]. The corresponding estimates of growth and maturity parameters could therefore depend on the relative stock compositions encountered by fisheries and sampling programs at any given time and place.

One major issue for bluefin tuna stock assessment is that, in addition to the mixed-stock structure of the Atlantic Ocean, there is further population structure within the Mediterranean [12]. Recent genetic research indicates that there is a discrete eastern Mediterranean population that is residential to the region [12]. These residential bluefin may be more productive than nomadic fish that move in and out of the Mediterranean Sea, potentially bolstering their capacity to withstand overfishing. Thus, it may be reasonable to consider a three-stock model to capture the additional mixed-stock dynamics. Considerable analytical work will be needed to capture how the violation of several assumptions could affect the reliability of MAST and other models in describing current and future population status.

New data and analytical techniques are revolutionizing our capacity to study the population structure and mixing of Atlantic bluefin tuna. The integration of multiple data types into a finer-scale spatial and temporal assessment of fish movement is a substantial advance in the development of tools for understanding bluefin tuna population dynamics. Incorporation of oceanographic data may enable tuning of models to discern seasonal aggregations in association with preferred ocean conditions. The data provide much needed biological information on how bluefin tunas utilize their entire range, and MAST allows us to synthesize these data. Many researchers have recognized the need to capture this new biological information in stock assessment models, and some have argued for the development of a management strategy evaluation (MSE) of Atlantic bluefin tuna fisheries [27]. The MAST model offers new directions for these cooperative efforts, and could be used as a reference model to simulate the performance of single-stock models, area-specific quotas, spatial management measures, and the interdependence of rebuilding the western and eastern stocks.

## Supporting Information

Figure S1 **Map of MAST spatial areas and electronic tag geolocations.**
(TIF)

Figure S2 **Annual catches by longline (LL), purse-seine (PS), bait boat (BB), and other (Oth) gears in (A) the Gulf of Mexico, (B) the Gulf of St. Lawrence, (C) the western Atlantic, (D) the eastern Atlantic, and (E) the Mediterranean Sea.**
(TIF)

Table S1 **Description of symbols and indices used in MAST**
(DOC)

Table S2 **Initialization of age-structured model assuming selectivity at age, natural mortality, age-specific fecundity, and Beverton-Holt recruitment**
(DOC)

Table S3 **Partial derivatives for the derivation of *B_0_* from *MSY* and *F_msy_***
(DOC)

Table S4 **Data, estimated parameters, and initial states**
(DOC)

Table S5 **State dynamics and observation model**
(DOC)

Table S6 **Objective function calculation**
(DOC)

Table S7 **Life-history parameters defining φ**
(DOC)

Table S8 **Electronic tag data state-transition equations in the MAST model**
(DOC)

Table S9 **Archival tag observation probabilities for state-space likelihoods in the MAST model for Atlantic bluefin tuna**
(DOC)

Table S10 **Summary of conventional tag cohorts of Atlantic bluefin tuna in the MAST model**
(DOC)

Table S11 **Summary of commercial CPUE data on relative abundance in the MAST model for Atlantic bluefin tuna**
(DOC)

Table S12 **Stock-composition data summary**
(DOC)

Table S13 **Summary of key MAST model output at the posterior mode for Atlantic bluefin tuna (A) base-case with time-invariant gear selectivity and normal reporting-rate priors; (B) estimated time-invariant gear selectivity and β(3,3) reporting-rate priors; (C) estimated time-varying gear selectivity and N(0.1,0.065) reporting-rate priors; (D) base-case with eastern age at 50% maturity at age 6; (E) base-case with bulk movement parameterization; and (F) single-stock model fit to estimated time-invariant gear selectivity. All projections to 2025 were run using constant catches of 1750 and 12,900 tonnes West and East, respectively.**
(DOC)

Table S14 **Estimated tag reporting rates of the MAST model for Atlantic bluefin tuna by scenario (A) base-case with time-invariant gear selectivity and normal reporting-rate priors; (B) estimated time-invariant gear selectivity and β(3,3) reporting-rate priors; (C) estimated time-varying gear selectivity and N(0.1,0.065) reporting-rate priors; (D) base-case with eastern age at 50% maturity at age 6; and (E) base-case with bulk movement parameterization. Case F is omitted in this table because mark-recapture data were not used for single-stock-model fitting.**
(DOC)

Text S1 (DOC)
