# Supplementary figures and images for: Atlantic Bluefin Tuna: A Novel Multistock Spatial Model for Assessing Population Biomass
Source: PLoS One. 2011 Dec 9;6(12):e27693. doi: 10.1371/journal.pone.0027693 (PMC3235089; doi:10.1371/journal.pone.0027693)

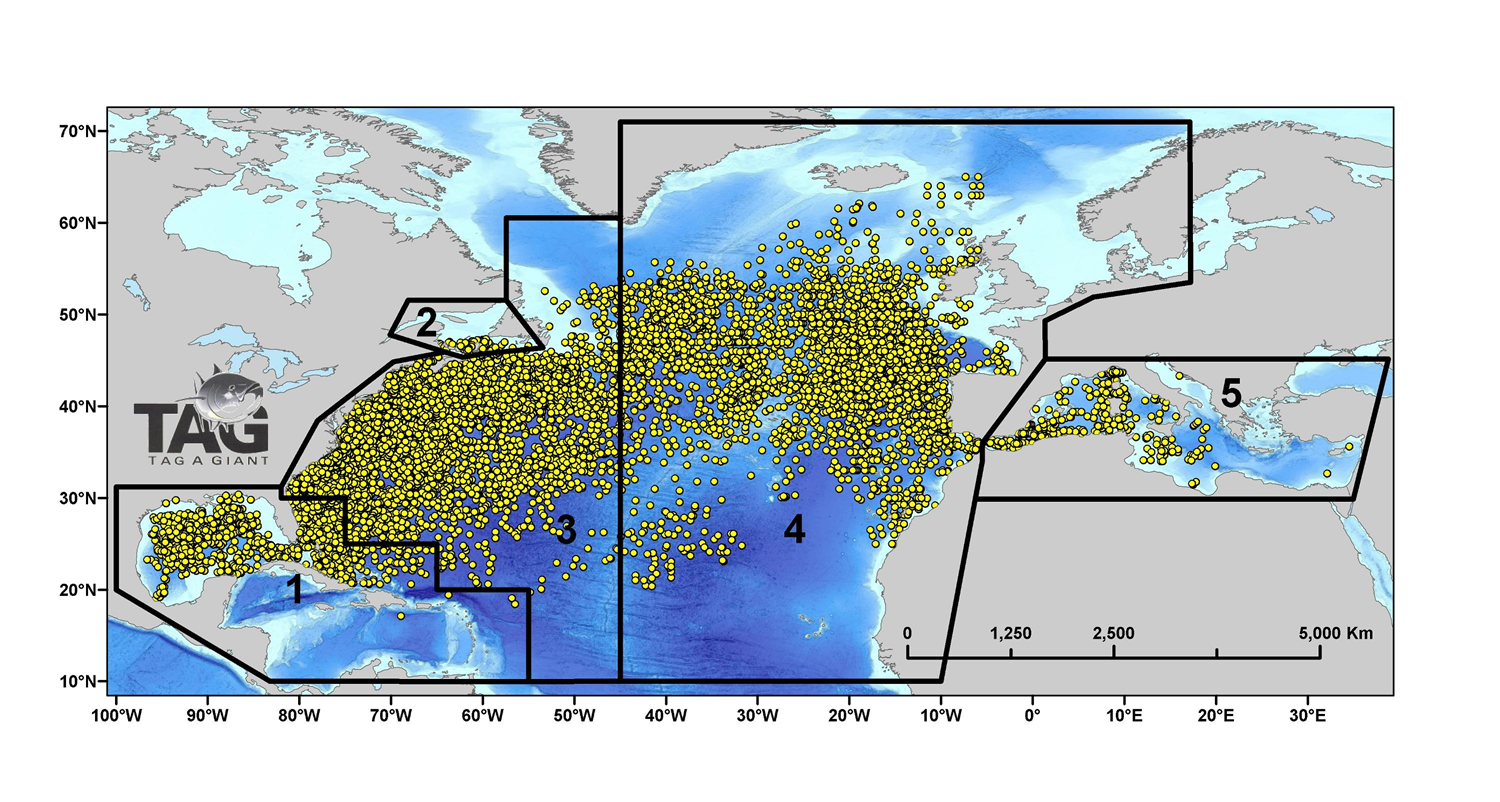

Supplement: Figure S1 — Map of MAST spatial areas and electronic tag geolocations. (TIF) [file pone.0027693.s001.tif]

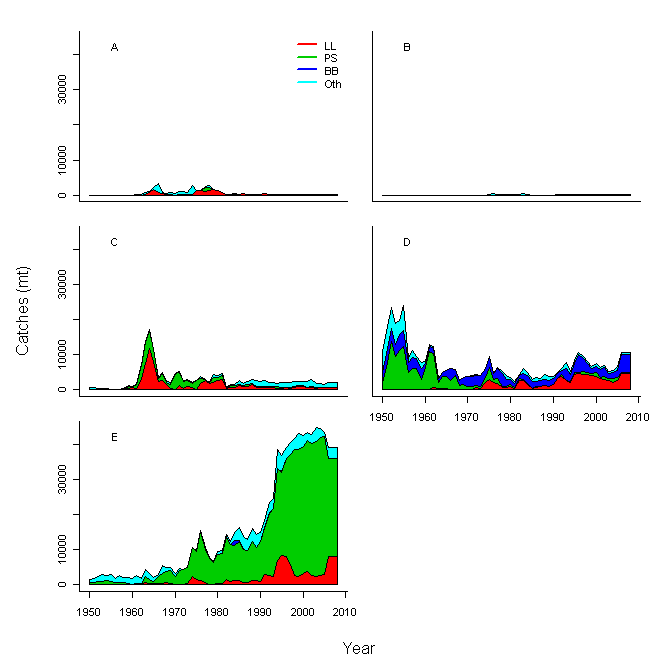

Supplement: Figure S2 — Annual catches by longline (LL), purse-seine (PS), bait boat (BB), and other (Oth) gears in (A) the Gulf of Mexico, (B) the Gulf of St. Lawrence, (C) the western Atlantic, (D) the eastern Atlantic, and (E) the Mediterranean Sea. (TIF) [file pone.0027693.s002.tif]
