# Supplementary material for: Atlantic Bluefin Tuna: A Novel Multistock Spatial Model for Assessing Population Biomass
Source: PLoS One. 2011 Dec 9;6(12):e27693. doi: 10.1371/journal.pone.0027693 (PMC3235089; doi:10.1371/journal.pone.0027693)
Supplement: Table S1 — Description of symbols and indices used in MAST (DOC) [file pone.0027693.s003.doc]

Table S1. Description of symbols and indices used in MAST

| **Indices** |  |
| --- | --- |
| *i,j,j,*$\mathop{j}\limits^{\sim}$  *t,qr,y*  *a,A,t,n_i_,sr*  *os,tt,s,h*  *natag,nptag*  *k* | Index for: stock, source area, destination area, spawning area  time, quarters, years  age, max age, time, number of areas, CPUE series  otolith sample, tag-type, tag-state, conventional tag cohort  number of archival tag tracks, pop-up satellite archival tracks  discrete electronic event type |
| **Estimated population parameters (θ)** |  |
| *MSY* | Maximum sustainable yield (kt) |
| *F_msy_* | Fishing rate to yield msy (yr^-1^) |
| *µ* | Matrix of movement parameters |
| *p_g_* | Probability of a geoposition |
| *σ_π_* | Standard deviation of pop-off quarter given programmed pop-off |
| ρ_c_ | Reporting rates conventional tags |
| *γ_g_* | Slope of gear selectivity ogive |
| *L_h_* | Length at half-selectivity |
| Τ | Over-dispersion for conventional mark-recapture likelihood |
| τ_2_ | Over-dispersion for proportions at age |
| **Life-history parameters** |  |
| *L_∞_* | Asymptotic size |
| *K* | Metabolic growth coefficient |
| *t_0_* | Theoretical time at zero length |
| *a,b* | Parameters for length-weight relationship |
| *L* | Predicted length |
| *a_h_, γ_fs_* | Age at half-maturity, slope of the maturity ogive |
| *f, w* | Fecundity, predicted weight |
| *Q* | Rate of conventional tag shedding |
| **Derived variables** |  |
| *B_0_* | Unfished steady-state biomass |
| *κ* | Recruitment compensation ratio |
| *R_e_* | Equilibrium age-0 recruitment |
| *R_0_* | Unfished steady-state recruitment |
|  | Survivorship unfished, fished |
|  | Eggs per recruit, unfished and fished |
|  | Vulnerable biomass per recruit, unfished and fished |
|  | Vulnerable biomass per recruit, available to the fishery |
| *α,β* | Beverton-Holt recruitment parameters |
| **State variables** |  |
| *N,V,E* | Numbers, vulnerable numbers, eggs |
| *B, VB* | Biomass, vulnerable biomass |
| *Bv* |  |
|  | Predicted logarithm of vulnerable biomass/numbers for CPUE |
| $\mathop{R^{'}}\limits^{.}$ | Predicted tag cohort numbers |
