# Supplementary material for: Atlantic Bluefin Tuna: A Novel Multistock Spatial Model for Assessing Population Biomass
Source: PLoS One. 2011 Dec 9;6(12):e27693. doi: 10.1371/journal.pone.0027693 (PMC3235089; doi:10.1371/journal.pone.0027693)
Supplement: Table S2 — Initialization of age-structured model assuming selectivity at age, natural mortality, age-specific fecundity, and Beverton-Holt recruitment (DOC) [file pone.0027693.s004.doc]

Table S1. Initialization of age-structured model assuming selectivity at age, natural mortality, age-specific fecundity, and Beverton-Holt recruitment

| **Life-history parameters** |
| --- |
| $\Phi_{i}=(L_{\infty,i},K_{i},t_{0,i},a_{i},b_{i},lh_{i},\gamma_{i})$ (1) |
| **Age-schedule information** |
| $l_{a,i}=L_{\infty,i}(1-\exp(-K_{i}(a-a_{0,i})))$ (2) |
| $w_{a,i}=a_{i}l_{a,i}^{b_{i}}$ (3) |
| $v_{a,i}=(1+\exp(\gamma_{g,t}(l_{a,i}-lh_{g,t})))^{-1}$ (4) |
| $f_{a,i}=w_{a,i}(1+\exp(\gamma_{f,i}(a-ah_{i})))^{-1}$ (5) |
| **Survivorship** |
| (6) |
| **Incidence functions** |
| (7) |
| (8) |
| (9) |
| **Derived variables** |
| (10) |
| (11) |
| (12) |
| (13) |
| (14) |
| (15) |
