# Supplementary material for: Atlantic Bluefin Tuna: A Novel Multistock Spatial Model for Assessing Population Biomass
Source: PLoS One. 2011 Dec 9;6(12):e27693. doi: 10.1371/journal.pone.0027693 (PMC3235089; doi:10.1371/journal.pone.0027693)
Supplement: Table S3 — Partial derivatives for the derivation of B0 from MSY and Fmsy (DOC) [file pone.0027693.s005.doc]

Table S1. Partial derivatives for the derivation of *B_0_* from *MSY* and *F_msy_*

| **Mortality and survival** |
| --- |
| $Z_{a,i}=M_{i}+F_{MSY,i}v_{a.i}$ (16) |
| $S_{a,i}=1-e^{-Z_{a,i}}$ (17) |
| **Partial derivatives for survivorship** |
| (18) |
| **Partial derivatives for incidence functions** |
| (19) |
| (20) |
|  |
| **Partial derivative for recruitment** |
| (21) |
