# Supplementary material for: Atlantic Bluefin Tuna: A Novel Multistock Spatial Model for Assessing Population Biomass
Source: PLoS One. 2011 Dec 9;6(12):e27693. doi: 10.1371/journal.pone.0027693 (PMC3235089; doi:10.1371/journal.pone.0027693)
Supplement: Table S4 — Data, estimated parameters, and initial states (DOC) [file pone.0027693.s006.doc]

Table S1. Data, estimated parameters, and initial states

| **Data** |  |
| --- | --- |
| *C_j,t_, I_j,sr,t_* | Catches, commercial CPUE |
| *A_j,t_* | Age-composition data |
|  |  |
| $\mathop{R_{h}^{'}}\limits^{.}$ | Released tags in cohort *h* |
| $x_{a,h,i,j,t}$ | Observed conventional recaptures |
| $Y_{i,t}$ | Observed electronic tag data |
| *Nos* | Number of GOM otoliths in a given sample |
| *Kos* | Total number of otoliths in a given sample |
| **Estimated parameters** |  |
| *θ_і_* | *MSY_i_, F_MSY,i_, ρ_j_, ω_it_,τ,lh, γ_g_* |
| **Initial states (t=1)** |  |
|  | Numbers in spawning area$\mathop{j}\limits^{\sim}$in first model year |
