# Supplementary material for: Atlantic Bluefin Tuna: A Novel Multistock Spatial Model for Assessing Population Biomass
Source: PLoS One. 2011 Dec 9;6(12):e27693. doi: 10.1371/journal.pone.0027693 (PMC3235089; doi:10.1371/journal.pone.0027693)
Supplement: Table S5 — State dynamics and observation model (DOC) [file pone.0027693.s007.doc]

Table S1. State dynamics and observation model

| **Estimated parameters** | **Description** |
| --- | --- |
| ***θ****_і_* | *MSY_i_, F_MSY,i_, ρ_j_, ω_it_,τ,lh, γ_g_* |
| (22) | Predicted catch used for solving for *F* |
| $N_{i,j',a+t,t+1,k}=\sum_{j} N_{i,j,a,t,}\mu_{i,a,t+1}(j,j')e^{-M_{i}+v_{i,a}F_{_{j,t}}}$ (23) | Numbers |
| $N_{j,a,t}=\sum_{i=1}^{2} N_{i,j,a,t}$ (24) | Total numbers |
| $R_{i,yr}=\frac{a_{i}E_{i,yr}}{1+\beta_{i,yr}}e^{\omega_{i,yr}}$ (25) | Recruits |
| $B_{j,a,t}=\sum_{i=1}^{2} N_{i,j,a,t}w_{i,a,t}$ (26) | Biomass |
| $V_{j,a,t}=\sum_{i=1}^{2} Nv_{i,j,a}$ (27) | Vulnerable numbers for the fishery |
| $VB_{j,a,t,g}=\sum_{i=1}^{2} Nv_{i,j,a,t,g}w_{i,a,t}$ (28) | Vulnerable biomass for the fishery |
| $V_{yr,sr}=\sum_{i} \sum_{qr(sr)} \sum_{j(sr)} \sum_{a(sr)} V_{a,i,j,q}$ (29) | Vulnerable survey numbers in each yr |
| $VB_{yr,sr}=\sum_{i} \sum_{qr(sr)} \sum_{j(sr)} \sum_{a(sr)} V_{a,i,j,q}w_{i,a}$ (30) | Vulnerable survey biomass |
| $P_{oto,os}=\frac{\sum_{t(os)} \sum_{a(os)} N_{i=1,t,j}}{\sum_{t(os)} \sum_{a(os)} \sum_{i} N_{i,t,j}}$ (31) | Otolith sample proportions *P_oto_* |
| (32) | Predicted age-composition proportions for multivariate logistic likelihood |
| $H_{i,j,h,a+t,t+1}=\begin{matrix} \mathop{R_{h,t}^{'}}\limits^{.} (D_{c,t}) & t=1 \\ \sum_{j} H_{i,j,a,t,h}\mu_{i,a}(j,j')e^{-M_{i}+\sum_{g} v_{i,a,g}F_{j,t}}(D_{c,t}) & t>1 \end{matrix}$ (33) | Predicted conventional tag cohort numbers |
| $\mathop{R}\limits^{.} _{i,j,a,t,h}=\rho_{j}H_{i,j,h,a,t}(1-e^{-v_{l}F_{t}})R_{a,h}^{-1}$ (34) | Predicted recaptures |
| (35) | Z statistics for CPUE data fitting |

Table S2. Objective function calculation

| **Log-likelihood functions, log(P(data|θ))** |  | **Data** |
| --- | --- | --- |
|  | (36) | CPUE |
| $L_{2}=\sum_{a} \sum_{h} \sum_{t} \begin{matrix} \log(\Gamma(x_{a,h,i,j,t}+r))-\log(\Gamma(r))-\log(\Gamma(x_{_{a,h,i,j,t}}+1)) \\ +r\log(r)+x_{_{a,h,i,j,t}}\log(P_{_{a,h,i,j,t}})-(r+x_{_{a,h,i,j,t}})*\log(r+P_{_{a,h,i,j,t}}) \end{matrix}$ | (37) | Conventional tag |
| $L_{3}=\sum_{n_{os}} \log\left(\begin{matrix} Nos \\ Kos \end{matrix}\right)+Kos\log(P_{oto})+(Nos-Kos)\log(1-P_{oto})$ | (38) | Otolith stock composition |
| $L_{4}=\sum_{tt} \sum_{t\in Y_{tt}} \log\left(P\left(Y_{tt,t-1}\right)P\left(y_{tt}\vert Y_{tt,t-1}\right)\right)\text{ }$ | (39) | Electronic tags |
|  | (40) | Age-composition data |
| **Priors, P(θ))** |  | **Distribution** |
| *MSY* | (41) | {Uniform(0.7,24), Uniform (10, 10000)}f |
| *F_msy_* | (42) | Normal(0.0611,0.067) Normal(0.069,0.015). |
| *µ* | (43) | Dirichlet(2,2) |
| *p_g_* | (44) | Beta(2,2) |
| *σ_π_* | (45) | Normal(1,5) |
| ρ_j_ | (46) | Normal(0.1,0.063) |
| Τ | (47) | Exponential |
