# Supplementary material for: Atlantic Bluefin Tuna: A Novel Multistock Spatial Model for Assessing Population Biomass
Source: PLoS One. 2011 Dec 9;6(12):e27693. doi: 10.1371/journal.pone.0027693 (PMC3235089; doi:10.1371/journal.pone.0027693)
Supplement: Table S6 — Objective function calculation (DOC) [file pone.0027693.s008.doc]

Table S1. Objective function calculation

| **Log-likelihood functions, log(P(data|θ))** |  | **Data** |
| --- | --- | --- |
|  | (36) | CPUE |
| $L_{2}=\sum_{a} \sum_{h} \sum_{t} \begin{matrix} \log(\Gamma(x_{a,h,i,j,t}+r))-\log(\Gamma(r))-\log(\Gamma(x_{_{a,h,i,j,t}}+1)) \\ +r\log(r)+x_{_{a,h,i,j,t}}\log(P_{_{a,h,i,j,t}})-(r+x_{_{a,h,i,j,t}})*\log(r+P_{_{a,h,i,j,t}}) \end{matrix}$ | (37) | Conventional tag |
| $L_{3}=\sum_{n_{os}} \log\left(\begin{matrix} Nos \\ Kos \end{matrix}\right)+Kos\log(P_{oto})+(Nos-Kos)\log(1-P_{oto})$ | (38) | Otolith stock composition |
| $L_{4}=\sum_{tt} \sum_{t\in Y_{tt}} \log\left(P\left(Y_{tt,t-1}\right)P\left(y_{tt}\vert Y_{tt,t-1}\right)\right)\text{ }$ | (39) | Electronic tags |
|  | (40) | Age-composition data |
| **Priors, P(θ))** |  | **Distribution** |
| *MSY* | (41) | {Uniform(0.7,24), Uniform (10, 10000)}f |
| *F_msy_* | (42) | Normal(0.0611,0.067) Normal(0.069,0.015). |
| *µ* | (43) | Dirichlet(2,2) |
| *p_g_* | (44) | Beta(2,2) |
| *σ_π_* | (45) | Normal(1,5) |
| ρ_j_ | (46) | Normal(0.1,0.063) |
| Τ | (47) | Exponential |
