# Supplementary material for: Atlantic Bluefin Tuna: A Novel Multistock Spatial Model for Assessing Population Biomass
Source: PLoS One. 2011 Dec 9;6(12):e27693. doi: 10.1371/journal.pone.0027693 (PMC3235089; doi:10.1371/journal.pone.0027693)
Supplement: Table S7 — Life-history parameters defining φ (DOC) [file pone.0027693.s009.doc]

Table S1. Life-history parameters defining Ф

| **Parameter** | **Symbol** | **Value for western stock** | **Value for eastern stock** |
| --- | --- | --- | --- |
| Asymptotic size | *L_∞_* | 304 cm | 304 cm |
| von Bertalanffy growth parameter | *K* | 0.098 yr^-1^ | 0.098 yr^-1^ |
| Age at theoretic zero length | *t_o_* | -0.15 years | -0.15 years |
| Age at half-maturity | *a_h_* | 12 | 4 (optional 6) |
| Slope of the maturity ogive | *γ_fs_* | 2 | 2 |
| Length-weight conversion coefficient | *A* | 2.86e-5 | 2.95e-5 <100 cm |
| Length-weight conversion exponent | *B* | 2.929 | 2.899 ≤100 cm  3.05 ≥101 cm |
| Natural mortality rate | *M* | 0.14 yr^-1^ | 0.14 yr^-1^ |
