# Supplementary material for: Atlantic Bluefin Tuna: A Novel Multistock Spatial Model for Assessing Population Biomass
Source: PLoS One. 2011 Dec 9;6(12):e27693. doi: 10.1371/journal.pone.0027693 (PMC3235089; doi:10.1371/journal.pone.0027693)
Supplement: Table S8 — Electronic tag data state-transition equations in the MAST model (DOC) [file pone.0027693.s010.doc]

Table S1. Electronic tag data state-transition equations in the MAST model

| **From tag state s_t_** | **To tag state s_t_** | **State transition probabilities p(st|Yt_-1_)** |
| --- | --- | --- |
| On fish in areas *V_i_* | On fish in areas *V_i_* | $P(s_{t+1}\vert Y_{t})=\sum_{j=V_{i}(1)}^{V_{i}(n_{i})} \mu(j,j')P(s_{t}\vert Y_{t-1})e^{-(M_{i,t-1}+v_{a,i,t-1}F_{j,t-1})}(1-D_{t-1})$ (48) |
| On fish in areas *V_i_* | Captured in fishing gear *g* in areas *V_i_* | $P(s_{t+1}\vert Y_{t})=\sum_{j=V_{i}(1)}^{V(n_{i})} \mu(j,j')P(s_{t}\vert Y_{t-1})e^{-(M_{i,t-1})}(1-e^{-M_{i,t-1}+v_{a,i,t-1}F_{j,t-1}})(1-D_{t-1})$ (49) |
| On fish in areas *V_i_* | Shed | $P(s_{t+1}\vert Y_{t})=\sum_{j=V_{i}(1)}^{V(n_{i})} \mu(j,j')P(s_{t}\vert Y_{t-1})e^{-(m_{i,t-1})}e^{-M_{i,t-1}+v_{a,i,t-1}F_{j,t-1}}D_{t-1}$ (50) |
| On fish in areas *V_i_* | Dead in areas *V_i_* | $P(s_{t+1}\vert Y_{t})=\sum_{j=V_{i}(1)}^{V(n_{i})} \mu(j,j')P(s_{t}\vert Y_{t-1})e^{-M_{i,t-1}+v_{a,i,t-1}F_{j,t-1}}(1-e^{-m_{i,t}})(1-D_{t-1})$ (51) |
| Dead | Dead | $P(s_{t+1}\vert Y_{t})=1$ (52) |
| Dead | Shed | $P(s_{t+1}\vert Y_{t})=0$ (53) |
| Shed | Shed | $P(s_{t+1}\vert Y_{t})=1$ (54) |
