# Supplementary material for: Atlantic Bluefin Tuna: A Novel Multistock Spatial Model for Assessing Population Biomass
Source: PLoS One. 2011 Dec 9;6(12):e27693. doi: 10.1371/journal.pone.0027693 (PMC3235089; doi:10.1371/journal.pone.0027693)
Supplement: Table S9 — Archival tag observation probabilities for state-space likelihoods in the MAST model for Atlantic bluefin tuna (DOC) [file pone.0027693.s011.doc]

Table S1. Archival tag observation probabilities for state-space likelihoods in the MAST model for Atlantic bluefin tuna

| **Tag state *s_t_*** | **Observation probabilities p(y_t_|s_t_) where *k*>0** | **Observation probabilities p(y_t_|s_t_) where *k*=0** |
| --- | --- | --- |
| Alive in areas 1 to *n_i_* | *p_tt_* | *1-p_tt_* |
| Captured in fishing gear *g* in area *k* | 1 | 0 |
| Shed | 0 | 1 |
| Dead | 0 | 1 |
