# Supplementary material for: Atlantic Bluefin Tuna: A Novel Multistock Spatial Model for Assessing Population Biomass
Source: PLoS One. 2011 Dec 9;6(12):e27693. doi: 10.1371/journal.pone.0027693 (PMC3235089; doi:10.1371/journal.pone.0027693)
Supplement: Table S10 — Summary of conventional tag cohorts of Atlantic bluefin tuna in the MAST model (DOC) [file pone.0027693.s012.doc]

Table S1. Summary of conventional tag cohorts of Atlantic bluefin tuna in the MAST model

| **Stock designation** | **Number of cohorts** | **Min release year** | **Max release year** | **Mean release age (yr)** |
| --- | --- | --- | --- | --- |
| Western stock | 125 | 1956 | 2008 | 11.6 |
| Eastern stock | 142 | 1966 | 2008 | 5.6 |
| Unknown | 1465 | 1954 | 2008 | 7 |
