# Supplementary material for: Atlantic Bluefin Tuna: A Novel Multistock Spatial Model for Assessing Population Biomass
Source: PLoS One. 2011 Dec 9;6(12):e27693. doi: 10.1371/journal.pone.0027693 (PMC3235089; doi:10.1371/journal.pone.0027693)
Supplement: Table S11 — Summary of commercial CPUE data on relative abundance in the MAST model for Atlantic bluefin tuna (DOC) [file pone.0027693.s013.doc]

Table S1. Summary of commercial CPUE data on relative abundance in the MAST model for Atlantic bluefin tuna

| **Survey** | **CPUE Code** | **Area** | **Composition** | **Gear code** | **Unit** | **Quarters** | **Max year** | **Min year** | **Min age class** | **Max age class** | **Fitted** | |
| --- | --- | --- | --- | --- | --- | --- | --- | --- | --- | --- | --- | --- |
| Canadian Gulf of St Lawrence | Can GSL | 2 | Gulf of Mexico | Other | Numbers | 3 | 2007 | 1981 | 14 | 30 | Yes | |
| Southwest Nova Scotia | Can SWNS | 3 | Mixed | Other | Numbers | 3 | 2007 | 1988 | 8 | 10 | Yes | |
| US Rod and Reel <145 ' | US RR<145 | 3 | Mixed | Other | Numbers | 3 | 1992 | 1980 | 2 | 6 | Yes | |
| US Rod and Reel 66-114; | US RR66-114 | 3 | Mixed | Other | Numbers | 3 | 2007 | 1993 | 3 | 4 | Yes | |
| US Rod and Reel 115-144 | US RR115-144 | 3 | Mixed | Other | Numbers | 3 | 2007 | 1993 | 5 | 6 | Yes | |
| US Rod and Reel >177' | US RR>177 | 3 | Mixed | Other | Numbers | 3 | 2007 | 1993 | 9 | 11 | Yes | |
| Japanese longline ICCAT Area 2 | JLL AREA2 2to9 | 3 | Mixed | Longline | Numbers | 1 2 3 4 | 2006 | 1976 | 3 | 10 | Yes | |
| Japanese longline ICCAT Area 3 | JLL Area 3 | 4 | Mixed | Longline | Numbers | 1 2 3 4 | 2006 | 1990 | 3 | 10 | Yes | |
| Japanese longline ICCAT Area 17 and 18 | JLL AREA 17+18 | 2 | Mixed | Longline | Numbers | 1 2 3 4 | 2006 | 1976 | 11 | 30 | Yes | |
| Japanese longline ICCAT Area 4 | JLL Area 4 | 4 | Mixed | Longline | Numbers | 1 2 3 4 | 2005 | 1990 | 11 | 30 | Yes | |
| Japanese longline Gulf of Mexico | JLL GOM | 1 | Gulf of Mexico | Longline | Numbers | 2 | 1981 | 1974 | 11 | 30 | Yes | |
| Larval zero inflated | LARVAL ZERO INFLATED 12 | 1 | Gulf of Mexico | NA | Biomass | 2 | 2007 | 1977 | 11 | 30 | Yes | |
| Larval Pennington | LARVAL PENNINGTON 13 | 1 | Gulf of Mexico | NA | Biomass | 2 | 2007 | 1977 | 11 | 30 | Yes | |
| US Pelagic longline | US PLL GOM REP Means | 1 | Gulf of Mexico | Longline | Numbers | 1 | 2007 | 1987 | 9 | 30 | Yes | |
| Tagging | Tagging | 1 | Gulf of Mexico | NA | Numbers | 1 2 | 1981 | 1970 | 1 | 4 | Yes | |
| US Rod and Reel >195' | USRR>195 | 3 | Mixed | Other | Numbers | 1 | 2001 | 1983 | 9 | 30 | Yes | |
| Morroccan Trap | MO Trap | 5 | Mediterranean | Other | Numbers | 2 3 | 2004 | 1998 | 11 | 30 | Yes | |
| Japanese longline MED | JPNLL MED | 5 | Mediterranean | Longline | Numbers | 2 3 | 2004 | 1975 | 11 | 30 | Yes | |
| Japanese longline MED E. Atl. | JPNLL MED+Area 5 | 4 5 | Mixed | Longline | Numbers | 2 3 | 2004 | 1975 | 11 | 30 | No | |
| Spanish bait boat | SPBB15-25 | 4 | Mixed | Bait boat | Numbers | 2 3 | 2004 | 1970 | 4 | 4 | Yes | |
| Spanish bait boat | SPBB All | 4 | Mixed | Bait boat | Numbers | 2 3 | 2004 | 1975 | 2 | 7 | No | |
| Spanish trap | SPTrap | 4 | Mixed | Other | Numbers | 2 3 | 2004 | 1981 | 11 | 30 | Yes | |
| Spanish bait boat | SPBB4 | 4 | Mixed | Bait boat | Numbers | 2 3 | 2007 | 1975 | 5 | 5 | Yes | |
| Spanish bait boat | SPBB5 | 4 | Mixed | Bait boat | Numbers | 2 3 | 2007 | 1975 | 6 | 6 | Yes | |
| Spanish bait boat | SPBB7-15 | 4 | Mixed | Bait boat | Numbers | 2 3 | 2004 | 1975 | 8 | 16 | Yes | |
| Spanish bait boat | SPBB1 | 4 | Mixed | Bait boat | Numbers | 2 3 | 2006 | 1975 | 2 | 2 | Yes | |
| Spanish bait boat | SPBB2 | 4 | Mixed | Bait boat | Numbers | 2 3 | 2007 | 1975 | 3 | 3 | Yes | |
| Spanish bait boat | SPBB3 | 4 | Mixed | Bait boat | Numbers | 2 3 | 2007 | 1975 | 4 | 4 | Yes | |
| Norwegian purse seine | Norway | 2 | Mixed | Purse seine | Numbers | 3 | 1986 | 1950 | 7 | 30 | Yes | |
| French | France | 4 | Mixed |  | Biomass | 3 | 1975 | 1952 | 7 | 30 | Yes | |
| Japanese longline Florida | Florida historic | 3 | Mixed | Longline | Biomass | 2 3 | 1971 | 1964 | 11 | 30 | Yes | |
| Japanese longline Brazilian | JLL Brazil historic | 1 | Gulf of Mexico | Longline | Numbers | 2 3 | 1970 | 1960 | 11 | 30 | | Yes |
