# Supplementary material for: Atlantic Bluefin Tuna: A Novel Multistock Spatial Model for Assessing Population Biomass
Source: PLoS One. 2011 Dec 9;6(12):e27693. doi: 10.1371/journal.pone.0027693 (PMC3235089; doi:10.1371/journal.pone.0027693)
Supplement: Table S12 — Stock-composition data summary (DOC) [file pone.0027693.s014.doc]

**Table S12**. Stock-composition data summary

| **Area** | **Ages** |  | **Stock numbers** | |
| --- | --- | --- | --- | --- |
|  | **Minimum** | **Maximum** | **Western** | **Eastern** |
| Mid-Atlantic Bight | 1 | 5 | 37 | 50 |
| Mid-Atlantic Bight | 6 | 10 | 31 | 24 |
| Mid-Atlantic Bight | 11 | 30 | 8 | 4 |
| Gulf of Maine | 11 | 30 | 68 | 4 |
