# Supplementary material for: Atlantic Bluefin Tuna: A Novel Multistock Spatial Model for Assessing Population Biomass
Source: PLoS One. 2011 Dec 9;6(12):e27693. doi: 10.1371/journal.pone.0027693 (PMC3235089; doi:10.1371/journal.pone.0027693)
Supplement: Table S13 — Summary of key MAST model output at the posterior mode for Atlantic bluefin tuna (A) base-case with time-invariant gear selectivity and normal reporting-rate priors; (B) estimated time-invariant gear selectivity and β(3,3) reporting-rate priors; (C) estimated time-varying gear selectivity and N(0.1,0.065) reporting-rate priors; (D) base-case with eastern age at 50% maturity at age 6; (E) base-case with bulk movement parameterization; and (F) single-stock model fit to estimated time-invariant gear selectivity. All projections to 2025 were run using constant catches of 1750 and 12,900 tonnes West and East, respectively. (DOC) [file pone.0027693.s015.doc]

Table S13. Summary of key MAST model output at the posterior mode for Atlantic bluefin tuna (A) base-case with time-invariant gear selectivity and normal reporting-rate priors; (B) estimated time-invariant gear selectivity and β(3,3) reporting-rate priors; (C) estimated time-varying gear selectivity and N(0.1,0.065) reporting-rate priors; (D) base-case with eastern age at 50% maturity at age 6; (E) base-case with bulk movement parameterization; and (F) single-stock model fit to estimated time-invariant gear selectivity. All projections to 2025 were run using constant catches of 1750 and 12,900 tonnes West and East, respectively.

| **Scenario** | **A** | **B** | **C** | **D** | **E** | **F** |
| --- | --- | --- | --- | --- | --- | --- |
| **Selectivity blocks** | 1 | 1 | 2 | 1 | 1 | 1 |
| **Reporting-rate priors** | N(0.1, 0.063) | Beta(3,3) | N(0.1, 0.063) | N(0.1,0.063) | N(0.1,0.063) | NA |
| **Movement parameterization** | Gravity | Gravity | Gravity | Gravity | Bulk transfer | None |
| **Leading parameters** | 66 | 66 | 68 | 66 | 132 | 6 |
| **Number of process error parameters** | 112 | 112 | 112 | 112 | 112 | 112 |
| **SSB/SSB_0_ West (%)** | 17 | 26 | 18 | 32 | 21 | 17 |
| **SSB/SSB_0_ East (%)** | 33 | 40 | 35 | 22 | 29 | 10 |
| **SSB_2008_/SSB_msy_ West (%)** | 38 | 53 | 42 | 70 | 55 | 101 |
| **SSB_2008_/SSB_msy_ East (%)** | 75 | 92 | 82 | 52 | 63 | 78 |
| **SSB_2025_/SSB_msy_ West (%)** | 43 | 70 | 69 | 89 | 160 | 230 |
| **SSB_2025_/SSB_msy_ East (%)** | 127 | 138 | 135 | 107 | 130 | 57 |
| **MSY West (kt)** | 3.87 | 3.76 | 3.6 | 3.52 | 3.99 | 4.033 |
| **MSY East (kt)** | 24.9 | 25.3 | 23.7 | 22.7 | 21.8 | 27.71 |
| **F_msy_ West (yr^-1^)** | 0.057 | 0.39 | 0.055 | 0.046 | 0.081 | 0.09 |
| **F_msy_ East (yr^-1^)** | 0.088. | 0.086 | 0.086 | 0.091 | 0.088 | 0.08 |
| **SSB_2025_/SSBmsy West (%)** | 43 | 70 | 69 | 89 | 160 | 230 |
| **SSB_2025_/SSBmsy East (%)** | 127 | 138 | 135 | 107 | 130 | 57 |
| **Objective function value** | 11024.0 | 11027.8 | 11020.0 | 11060.3 | 11092.8 | 511.82 |
