# Supplementary material for: Atlantic Bluefin Tuna: A Novel Multistock Spatial Model for Assessing Population Biomass
Source: PLoS One. 2011 Dec 9;6(12):e27693. doi: 10.1371/journal.pone.0027693 (PMC3235089; doi:10.1371/journal.pone.0027693)
Supplement: Table S14 — Estimated tag reporting rates of the MAST model for Atlantic bluefin tuna by scenario (A) base-case with time-invariant gear selectivity and normal reporting-rate priors; (B) estimated time-invariant gear selectivity and β(3,3) reporting-rate priors; (C) estimated time-varying gear selectivity and N(0.1,0.065) reporting-rate priors; (D) base-case with eastern age at 50% maturity at age 6; and (E) base-case with bulk movement parameterization. Case F is omitted in this table because mark-recapture data were not used for single-stock-model fitting. (DOC) [file pone.0027693.s016.doc]

Table S14. Estimated tag reporting rates of the MAST model for Atlantic bluefin tuna by scenario (A) base-case with time-invariant gear selectivity and normal reporting-rate priors; (B) estimated time-invariant gear selectivity and β(3,3) reporting-rate priors; (C) estimated time-varying gear selectivity and N(0.1,0.065) reporting-rate priors; (D) base-case with eastern age at 50% maturity at age 6; and (E) base-case with bulk movement parameterization. Case F is omitted in this table because mark-recapture data were not used for single-stock-model fitting.

| **Scenario** | **GOM** | **GSL** | **WATL** | **EATL** | **MED** |
| --- | --- | --- | --- | --- | --- |
| A | 0.060 | 0.092 | 0.217 | 0.087 | 0.077 |
| B | 0.098 | 0.284 | 0.799 | 0.161 | 0.094 |
| C | 0.032 | 0.059 | 0.381 | 0.097 | 0.074 |
| D | 0.048 | 0.070 | 0.442 | 0.137 | 0.057 |
| E | 0.025 | 0.101 | 0.303 | 0.153 | 0.058 |
