# Supplementary material for: Atlantic Bluefin Tuna: A Novel Multistock Spatial Model for Assessing Population Biomass
Source: PLoS One. 2011 Dec 9;6(12):e27693. doi: 10.1371/journal.pone.0027693 (PMC3235089; doi:10.1371/journal.pone.0027693)
Supplement: Text S1 — (DOC) [file pone.0027693.s017.doc]

Supporting Information

In tables below we summarize each of the four main **M**ulti Stock **A**ge **S**tructured **T**ag Integrated assessment (MAST) model components: 1) initialization of the model; 2) updating state variables; 3) relating state variables to observations; 4) evaluating the probability of the model given the data.

S1 Description of model symbols and indices (components 1-4)

S2 Model initialization equations (component 1)

S3 Partial derivatives for the derivation of initial biomass, *Bo,* from maximum sustainable yield, *MSY,* and the optimal fishing mortality, *F_MSY_* (component 1)

S4 Data, estimated parameters and initial states (components 1 and 4)

S5 State dynamics (component 2) and observation model (component 3)

S6 Statistical objective function (component 4)

S7 Life-history parameters (components 1-4)

S8 Electronic tag state transitions (component 4)

S9 Archival tag observation probabilities (component 4)

We summarize the input data as well as describe model scenarios and some results in Tables S10-12 and S13-S14, respectively.

**Model Description**

*Initialization*

Initial numbers and biomass were derived from the leading parameters, maximum sustainable yield (*MSY*) and optimal fishing mortality rate (*F_msy_*), by (1) beginning with the equilibrium yield equation; (2) differentiating this function with respect to *F_msy_*; (3) setting this equation equal to 0; and (4) solving for the Beverton-Holt recruitment parameter *κ* (see Table S4). While others have used a similar approach , MAST includes a version that assumes an instantaneous fishing mortality rate . We outline the mathematics behind this derivation in Tables S2 and S3.

In practice, many fisheries data sets do not have enough contrast to reliably estimate scaling and productivity parameters , such as *MSY* and *F_msy_.* In such cases, prior probability distributions for these parameters must be used to facilitate parameter estimation. Catches relative to *MSY* and current fishing mortality rate relative to *F_msy_* are key performance measures that determine whether or not a particular fish stock is overexploited. Defining the model in terms *MSY* and *F_msy_* makes transparent the influence of priors on these quantities . The assumed values for the growth, maturity, and natural mortality rate parameters that define age schedules are given in Table S7

*State Dynamics*

The state dynamics for the MAST model are given in Table S5. MAST predicts fish numbers *N* in age-class *a*, of stock *i,* in each area *j',* at time *t+1,* to be the sum of all surviving fish at time *t* that move to (or stay in) *j',* from area *j,* according to the movement transition matrix *μ* (see Table S5). We model natural and fishing mortality as occurring simultaneously. In addition, MAST is conditioned on total catches by area. The fishing mortality rate *F* in each quarter is determined by solving the Baranov catch equation using the observed vulnerable landings and the estimated vulnerable biomass (Eq. 29, Table S5). The catch equation is numerically solved for *F* by the Newton-Rhapson iteration. In the second quarter of every year, fish move to their respective spawning areas (Gulf of Mexico for the western stock and the Mediterranean for the eastern stock) according to their stock-specific age-at-maturity schedules (Table S7).

The gravity models used to populate movement matrices *µ* are well developed in the geography literature and have been applied to some aquatic dispersal models and spatial fisheries models to describe fleet and fish movement between areas of differing relative attractiveness. The gravities of different areas for any population of interest can be modeled as mathematical functions of covariates such as area size, distance between areas, opportunities, and competition . Expressing gravities in terms of variables such as tuna density and food availability is a promising avenue for future research, in which other data such as ocean conditions could be used. However, we currently do not apply any functional form for the migration probabilities.

An alternative to using the gravity model parameterization is to parameterize movement in terms of diffusion or bulk transfer. With this approach, all *n_i_*-1 elements are allowed to vary except the *n_i_^th^* element, which is given by the sum of the other logit-transformed elements:

$\mu_{j,j'}=\frac{e^{u_{j,j'}}}{\sum_{j'} e^{u_{j,j'}}}$, where *u_j,1_* = 0 and all other *u_j,j’_* terms are estimated.

During model development, we considered several alternative gravity and bulk-transfer model versions that assumed movement rates were equal across ages and seasons; these models, however, would not converge using this compressed parameterization. Defining different movement matrices for more than two age-groups resulted in too many parameters to be estimated. We therefore confined our exploration of movement parameterization options to two age-groups for which movement parameters were assumed to be similar to those for ages 0–7 and 8+. We assumed that nonspawning fish were distributed as they would have been in nonspawning quarters by a set of estimated movement parameters for age-groups 0–7 and 8+. We used the gravity model as the base-case, and we explored the model’s sensitivity to the bulk movement parameterization, as shown below.

*Discrete State-Space Models for Electronic Tag Data*

We assumed that archival and pop-up tags have a recapture history of the form *Y_t_*={*y_1_*,*y_2_*...*y_s_*}, where for each possible sampling date, an observation *y_t_*=0 denotes no observation in state *s,* and *y_t_>*0 denotes at least one observation. We calculated the likelihood *P(Y_t_)* of each tag track using a recursive method in which the probability of the tag track observations up to time *t* *P(Y_t_)* was represented as

*P*(*Y_t_*)*=P*(*Y_t-1_*)*P*(*y_t_|Y_t-1_*)*.* (1)

For any given tag track, the probability of an event *k* (geolocation, pop-off, or recapture event), in a recapture history *P(y_t_|Y_t-1_),* can be written as

*P*(*y_t_|Y_t-1_*)*=*Σ*_s_P*(*s_t_|Y_t-1_*)*P*(*y_t_|s_t_,Y_t-1_*), (2)

where *s_t_* represents possible tag states (*s_t_*={on live fish in locations 1-*n*, shed from the fish, on a dead fish, etc.}) and *P*(*y_t_|s_t_,Y_t-1_*) is the observation probability of *y_t_* given that the fish is in state *s_t_*. Representation for *P*(*y_t_|Y_t-1_*) in terms of states *s_t_* expresses the problem of calculating it as two simpler problems—calculation of location state probabilities *P*(*s_t_|Y_t-1_*) and observation probabilities *P*(*y_t_|s_t_*) for that tag type (Table S9).

Seasonal and age dependencies of movement rates are incorporated in the estimation by assigning each fish of stock *i* an apparent age-at-first-capture based on its length. Each fish can then be assigned to a particular age-group whose movement parameters are assumed to be identical within that group.

*F_msy_ Prior Simulation*

For the model to converge, either fixed values of *F_msy_* or prior probability distributions were required. To simulate priors for *F_msy_*, we used compiled mean and standard deviation values on the standardized slope of the origin for stock-recruit functions fitted to data for populations of Scombridae , excluding Atlantic bluefin tuna. We then simulated *F_msy_* priors based on an assumed *B_0_* of unity, and assumed mean growth, mortality, and maturity parameters. The mortality and maturity parameters were sampled assuming normal distributions and means given in Table S7, and assumed coefficients of variations of 0.1. For the purposes of simulating the prior, selectivity parameters *l_h_* and *γ* were assumed to be fixed at *l_h_*=100 and *γ*=0.5.

**Data**

*Satellite Tag Data*

Satellite tag end-point positions were determined upon release by using the Doppler shift of radio transmissions to ARGOS satellites. For electronic tag types, daily estimates of longitude for tagged fish while at liberty were derived from algorithms based on tag measurements of light levels with manufacturer programs as previously described . Daily estimates of latitude were improved with sea-surface temperatures as previously described . Missing positions in the tag tracks arising from factors such as cloud cover in the satellite observations of temperatures that were used to estimate latitude were imputed via linear interpolation. For the present analysis, all geolocations for each fish were distilled in quarterly time intervals and the five MAST areas based on the modal area occupied at each quarter. We used 220 pop-off satellite and 122 archival tag tracks with complete records extending from 1996 to 2008.

*Stock and Age Assignment for Tag Data*

Both conventional and electronic tag data were classified as eastern, western, or unknown stock of origin on the basis of area of visitation. All fish that visited the Gulf of Mexico or Gulf of St. Lawrence were assumed to be of western-stock origin, an assumption supported by otolith and genetic data We assumed that fish that visited the Mediterranean Sea were of eastern-stock origin, supported by current genetic and otolith data sets. All other fish were considered to be of unknown stock of origin.

Age assignments to all tagged fish were made using the same growth curves used to derive the catch-at-age input for ICCAT’s VPA . We used growth parameters estimated by in the east and in the west, because these growth curves are considered representative of the populations vulnerable to capture for those stocks and therefore appropriate for the assignment of ages from lengths. However, we used Neilson and Campana’s von Bertalanffy growth parameter estimates to predict body size for both western and eastern stocks in the population dynamics model described below, because this analysis is the only one for bluefin tuna that uses validated age data.

Conventional tag retentions *D_c,t_* are modeled according to , assuming decreasing shedding rates over time with no immediate tag shedding. The probability of retaining a tag at time *t,* *D(t),* is given as *D(t)=e^-Q(t)^*, and the rate of shedding at time *t* is *Q(t)=*[*dλ*/(*d+λt)*]*^d^*. We assumed values for *d*=0.22 and *λ*=1.95 using means across all tagging experiments fitted using model 3 of Kurota *et al.* . Age proportion data were fitted using multivariate logistic likelihoods (Tables S5 and S6). The likelihood function for the otolith stock-composition data was binomial (Table S6).

*Catch Data*

The difference between the inflated and observed eastern catches was assumed to come entirely from purse seines in the Mediterranean Sea. For 1999–2009, we assumed that unreported and assumed catches were distributed within years according to the proportion of the annual catch, by quarter, reported in years before illegal and unreported fishing occurred in 1998.

*Parameter Estimation*

The nonlinear optimization procedure that was applied to estimate model parameters is described in the main text. The equations describing the objective function that is minimized are listed in Table S6.

*Sensitivity Cases*

We examined the sensitivity of the base-case results across a suite of alternative model parameterizations and reporting-rate-prior distributions. We evaluated the effects of using this informative prior by applying a much less restrictive prior for conventional tag reporting rates, *beta*(3,3) (Table S13, scenario B). We divided gear selectivities into pre- and post-1991 because early bluefin tuna catches were known to have targeted smaller fish (scenario C). Scenario D assumes the same parameter values as the base-case, but with an eastern stock age at 50% maturity at age 6. Scenario E was the base-case with bulk movement parameterization. Finally, scenario F was a single-stock, single-time block selectivity *F_msy_*/*MSY* formulation that was fitted to the same CPUE and age-composition data as the mixed models, but without using mark-recapture data.

In the single-stock case, we treated all CPUE, catch, and catch-at-age data coming from areas 1–3 as western stock, and data from areas 4–5 as eastern stock. Life-history parameters for each stock were assumed to be those listed for eastern and western stocks in Table S7. Finally, we compared the model results to reconstructed spawning stock biomasses for western and eastern stocks using the ICCAT VPA assessment. For the eastern stock, we used the ICCAT assessment inflated catch-at-age case (g) using the ICCAT 2008 assessment (4) run 14. For the western stock, we included the ICCAT 2008 western base case (g), as well as the case 9 sensitivity run excluding the Gulf of St. Lawrence (GSL) index (h)(ICCAT 2008).

*Sensitivity Analyses*

Across all sensitivity scenarios, the MAST model suggests that western bluefin tuna constitute a much smaller stock than the eastern stock, with lower estimated *MSY*s under all scenarios (Table S13). MAST results also suggest that the western stock has consistently lower *F_msy_* than the eastern stock (Table S13).

Reference points, current and future biomasses, and depletion levels differed between model options (Table S13). Across all MAST scenarios and the single-stock statistical catch-at-age model, the variability of depletion levels was large, ranging between 17% and 26% for the western stock and between 10% and 40% for the eastern stock. In all mixed-stock models examined, the current western-stock spawning stock depletion as reflected in the *SSB_2008_*/*SSB_msy_* ratio was predicted to be between 38% and 70% (Table S13, A–E). The spawning stock biomass was predicted to be at levels that produce *MSY* using the single-stock statistical catch-at-age model (Table S13, F). The assumption of less informative reporting-rate priors resulted in much lower estimates of depletion levels and higher estimates of stock size relative to the unfished state (Table S13, B). All models showed that the eastern stock was overfished, with *SSB_2008_*/*SSB_msy_* ratios ranging between 52% and 92% (Table S13, A–E). Importantly, the predicted performance of rebuilding options was also highly variable between different model formulations. The model’s predictions at the posterior mode showed that the western stock would only recover to levels that would produce *MSY* by 2025 using the bulk-transfer and single-stock statistical catch-at-age models (Table S13, E and F). Otherwise, it was predicted to be well below *B_msy_*, with *SSB*/*SSB_msy_* values ranging between 43% and 89% (Table S13, A–D). The eastern stock was predicted to recover by 2025 in all scenarios except the single-stock statistical catch-at-age model (Table S13). MAST model results are sensitive to reporting-rate-prior assumptions (Table S13). When a less informative prior (*beta*(3,3)) was used, reporting rates were estimated to be much higher across all areas (Table S14), approaching unrealistically high values such as 80% in the western Atlantic. Corresponding to these reporting rate differences, estimated biomasses relative to reference points were also higher and rebuilding times faster (Table S13).

**References**

1. Mace PM, Doonan IJ (1988) A generalized bioeconomic simulation model for fish population dynamics. NZ. Fish. Assess. Res. Doc. 88/4.

2. Schnute JT, Kronlund AR (1996) A management oriented approach to stock recruitment analysis. Can. J. Fish. Aquat. Sci. 53: 1281-1293.

3. Forrest RE, Martell SJD, Melnychuk MC, Walters CJ (2008) An age-structured model with leading management parameters, incorporating age-specific selectivity and maturity. Can J Fish Aquat Sci 65: 286-296.

4. Martell SJD, Pine WE, Walters CJ (2008) Parameterizing age-structured models from a fisheries management perspective. Can J Fish Aquat Sci 65: 1586-1600.

5. Ludwig D, Hilborn R (1983) Adaptive probing strategies for age structured fish stocks. Can J Fish Aquat Sci 40: 559-569.

6. Thomas RW, Huggett RJ (1980) Modeling in geography. Totowa, New Jersey: Barnes & Noble. 321 p.

7. Vries JJD, Nijkamp P, Rietveld P (2001) Alonso 's Theory of Movements : Developments in Spatial Interaction Modeling. J Geograph Syst 3: 233-256.

8. Bossenbroek JM, Kraft CE, Nekola JC (2001) Prediction of long-distance dispersal using gravity models: zebra mussel invasion of inland lakes. Ecol App 11: 1778-1788.

9. Martell SJD, Essington TE, Lessard B, Kitchell JF, Walters CJ, et al. (2005) Interactions of productivity, predation risk, and fishing effort in the efficacy of marine protected areas for the central Pacific. Can J Fish Aquat Sci 62: 1320-1336.

10. Walters CJ, Bonfil R (1999) Multispecies spatial assessment models for the British Columbia groundfish trawl fishery. Can J Fish Aquat Sci 56: 601-628.

11. Myers RA, Bowen KG, Barrowman NJ (1999) Maximum reproductive rate of fish at low population sizes. Can J Fish Aquat Sci 56: 2404-2419.

12. Block BA, Dewar H, Boustany A, Blackwell SB, Seitz A, et al. (2001) Migratory movements, depth preferences, and thermal biology of Atlantic bluefin tuna. Science 293: 1310-1314.

13. Block BA, Teo S, Walli A, Boustany A, Stokesbury MJW, et al. (2005) Electronic tagging and population structure of Atlantic bluefin tuna. Nature 434: 1121-1123.

14. Rooker JR, Secor DH, De Metrio G, Schloesser R, Block BA, et al. (2008) Natal homing and connectivity in Atlantic bluefin tuna populations. Science 322: 742-744.

15. Boustany AM, Reeb CA, Block BA (2008) Mitochondrial DNA and electronic tracking reveal population structure of Atlantic bluefin tuna (*Thunnus thynnus*). Mar Biol 156: 13-24.

16. Carlsson J, McDowell JR, Carlsson JEL, Graves JE (2007) Genetic identity of YOY bluefin tuna from the eastern and Western Atlantic spawning areas. J Hered 98: 23-28.

17. Riccioni G, Landi M, Ferrara G, Milano I, Cariani A, et al. (2010) Spatio-temporal population structuring and genetic diversity retention in depleted Atlantic bluefin tuna of the Mediterranean Sea. Proc Natl Acad Sci USA 107: 2102-2107.

18. ICCAT (2008) Report of the 2008 Atlantic bluefin tuna stock assessment session. Madrid. 247 p.

19. Cort JL (1991) Age and growth of bluefin tuna in the Northeast Atlantic. Col Vol Sci Pap ICCAT 35: 213-230.

20. Turner SC, Restrepo VR (1992) A review of the growth rate of West Atlantic bluefin tuna, Thunnus thynnus, estimated from marked and recaptured fish. Col Vol Sci Pap ICCAT 42: 170-172.

21. Neilson JD, Campana SE (2008) A validated description of age and growth of western Atlantic bluefin tuna (*Thunnus thynnus*). Can J Fish Aquat Sci 65: 1523-1527.

22. Hampton J, Kirkwood GP (1981) Tag Shedding by Southern bluefin tuna *Thunnus maccoyii*. Fish Bull 88: 313-321.

23. Kurota H, Mcallister MK, Lawson GL, Nogueira JI, Teo SLH, et al. (2009) A sequential Bayesian methodology to estimate movement and exploitation rates using electronic and conventional tag data : application to Atlantic bluefin tuna (*Thunnus thynnus*). Can J Fish Aquat Sci 66: 321-342.

24. Schnute JT, Richards LJ (1995) The influence of error on population estimates from catch-at-age models. Can. J. Fish. Aquat. Sci 52: 2063-2077.
